# The Institutional Worker

**Published:** 1912-03-23

**Authors:** 


### INSTITUTIONAL WORKER

**Being a Special Supplement to "The Hospital"**

### Editor's Notices.

**Contributions:**

Contributions should be written, or preferably typed, on one side of the paper only, and all articles sent in are accepted upon the distinct understanding that they are forwarded to THE HOSPITAL only.

The Editor cannot undertake to return MSS. not used, but when a stamped directed envelope is enclosed the MSS. may be returned if a special request is made.

Accepted articles and paragraphs of news will be paid for after publication at the scale rate.

**Address:**

To prevent delay all contributions and letters on editorial business must be addressed exclusively to the Editor, "The Hospital," 29 Southampton Street, Strand, London, W.C. It is important that this regulation shall be strictly observed.

**Correspondence:**

Correspondence on all subjects is invited. The name and address of correspondents must be given as a guarantee of good faith, but not necessarily for publication.

**Special Articles:**

Special articles are invited, and questions, inquiries, and paragraphs upon all matters relating to the work, administration, and management of general, special, mental, fever, cottage and convalescent hospitals, sanatoria, homes, institutions, societies and organisations for the treatment or care of the sick, injured and dependants of all classes. Every matter affecting the interests and welfare of the staff of all grades working in these institutions will receive special consideration.

**Administrative Medicine, Research and Items of News:**

Special terms will be paid for approved articles on subjects in this wide field by experts who have made some section of it their special study and interest. We wish to give prominence to every movement tending to advance accuracy of diagnosis, efficiency in treatment, and the development of modern methods to eradicate disease and promote the welfare of the suffering.

**A Bureau of Information:**

We invite inquiries and applications for information and help in providing for that numerous class of sufferers who require special care or house-room which they cannot obtain in their own homes or secure for themselves. We cannot, however, prescribe, or recommend practitioners.

**Books for Review:**

Publishers are particularly requested to send advance proofs of any new books of importance whenever possible, as the Editor has made arrangements to publish immediate reviews on a new plan.

**Photographs, Plans, Blocks, and Illustrations:**

It is requested that wherever possible MSS. may be accompanied by illustrations in any of the above forms. The name of the sender and of the article to which it belongs should be written on the back of each photograph, drawing, or block for purposes of identification.

**Local Papers:**

Newspapers containing reports or news paragraphs should be marked and addressed to the Sub-Editor.

**The Coupon System:**

A coupon will be found at the bottom of the third inside page of the cover each week. This coupon must be attached to every question or inquiry to which an answer is desired in THE HOSPITAL.

### Many Vacant Appointments.

**To the Worker:**

"THE INSTITUTIONAL WORKER" affords exceptional facilities to those seeking appointments in any grade or department of the Institutional, Health, and Sanitary Services. In this section a large number of vacancies in Hospitals, Infirmaries, and Institutions of every description throughout the United Kingdom are announced every week. Should you not find a post suited to your needs we would remind you that a column is reserved to those seeking re-engagement, and an announcement in this portion will enable you to bring your requirements prominently before the Executive Officials of every class of Institution, as well as those responsible for Poor Law and Municipal administration.

**To Institution Authorities:**

THE officials controlling institutions will also find THE INSTITUTIONAL WORKER of the greatest practical utility when a vacancy occurs upon their staffs, as an announcement in its columns secures the widest publicity among those trained to Institutional Work, and through its agency it is possible to obtain the most efficient officers in every section of Institutional Life. In these circumstances particular attention is drawn to the fact that SPECIAL RATES are allowed to those Institutions that agree for one year to send all their public notices for insertion in this section of THE HOSPITAL.

### Manager's Notices.

**Letters relating to the publication, sale, and advertising departments of "The Hospital" must be addressed to The Manager, "The Hospital," The Hospital Building, 28 and 29 Southampton Street, London, W.C.**

Subscriptions may begin at any time, and are payable in advance. Cheques and Post-Office orders should be crossed London County and Westminster Bank, Covent Garden branch, and made payable to the Manager of THE HOSPITAL.

RATES OF SUBSCRIPTION (payable in advance).

| | | |
|---|---|---|
| UNITED KINGDOM. | | |
| Three Months (including Postage) | ... ... ... ... | 2s. 0d. |
| Six Months | do. ... ... ... ... | 4s. 0d. |
| Twelve Months | do. ... ... ... ... | 6s. 6d. |
| FOREIGN AND COLONIAL. | | |
| Three Months (including Postage) | ... ... ... ... | 2s. 6d. |
| Six Months | do. ... ... ... ... | 5s. 0d. |
| Twelve Months | do. ... ... ... ... | 8s. 8d. |

**Advertisements:**

To ensure insertion, all advertisements for THE HOSPITAL must reach the Manager not later than Wednesday morning in each week. For scale of charges see pages vii and 2.

**Remittances:**

**It is especially requested that remittances be made by cheque or Postal Order, and in halfpenny stamps only when the amount is under sixpence.**

### OFFICIAL APPOINTMENTS VACANT.

### Poor-Law Vacancies.

Assistant Female Cook (£24), Bristol Guardians. The C. to G., Bristol. March 23.

Attendant for Children's Homes (12s. and rations weekly, non-resident), Bermondsey Guardians. Mr. E. Pitts Fenton, C. to G., 283 Tooley Street, S.E. March 25.

Assistant Labour Master (£30), Wandsworth Union. Mr. F. W. Piper, C. to G., St. John's Hill, Wandsworth, S.W. March 27.

Assistant Matron (£40), Wandsworth Union. Mr. F. W. Piper, C. to G., St. John's Hill, Wandsworth, S.W, March 27.

Assistant Nurse (£28), Bromley Union. G. Haslehurst, C. to G., Union Offices, Park House, Bromley, Kent. March 25.

Assistant Nurse (£28), West London District Schools, Ashford, Middlesex. F. G. Beeching, Clerk. March 29.

Assistant Nurse (£25), Hartismere Union. H. Warnes, C. to G., Union Offices, Eye, Suffolk. March 29.

Assistant Nurse (£20), Falmouth Union. R. N. Rogers, C. to G., Union Offices, Falmouth. March 26.

Assistant Nurse (£26), Newmarket Union. Mr. S. J. Ennion, C. to G., Newmarket. March 27.

Assistant Nurse (£30), Llanelly Union. D. C. Edwards, C. to G., Union Offices, Llanelly. April 8.

Assistant Nurses (2) (£18), Holborn Union. Matron of the Workhouse, Mitcham, Surrey.

Assistant Schoolmistress (£60), Walsall and West Bromwich School District. Mr. A. H. Ward, Clerk to the Managers, West Bromwich. March 23.

Barber-Bath Attendant (£30), Preston Union. Mr. J. Clarke, C. to G., Preston. March 28.

Boiler Fireman (£30), Preston Union. Mr. J. Clarke, C. to G., Preston. March 28.

Case-Paper Clerk (£65), Burton-upon-Trent Union. Mr. C. F. Chamberlin, C. to G., Burton-on-Trent. March 23.

Charge Nurse (£32), Bedford Union. W. Payne, C. to G., 115 High Street, Bedford. March 30.

Charge Nurses (£35), Carlisle Union. Mr. H. B. Lonsdale, C. to G., Carlisle. March 25.

Children's Attendant (£18), Bedford Union. Mr. W. Payne, C. to G., Bedford. March 30.

Children's Nurse (£30), Merthyr Tydfil Union. Mr. F. T. James, C. to G., Merthyr Tydfil. March 28.

Cook and Assistant Matron (£20), Goole Union. Mr. R. Haldenby, Clerk to the Children's Committee, Bank Chambers, Goole. March 26.

Female Assistant Infirm Attendant (£20), Bethnal Green Guardians. Matron of the Workhouse, Waterloo Road, Victoria Park, N.E.

Female Attendant (£25), Devizes Union. Mrs. Fear, Matron of the Workhouse, Devizes.

Female Cook (£30), Colchester Guardians. Mr. C. E. White, C. to G., Colchester. March 25.

Female General Attendant (£23), Fulham Guardians. Mr. E. J. Mott, C. to G., 129 Fulham Palace Road, W. March 25.

Female Superintendent of Scattered Homes (£75), Female Clerk to Ditto (£30), Brentford Union. Mr. W. Stephens, C. to G., Isleworth, W. March 26.

Headmaster's Clerk and Storekeeper (£30), Islington Guardians. Headmaster of the Schools, Hornsey, N.

Head Sister and Superintendent Nurse (£35), Ashton-under-Lyne Union. G. H. Partington, C. to G., Poor Law Offices, Ashton-under-Lyne. March 26.

Hospital Cook (Female) (£27), Burton-upon-Trent Union. Mr. C. F. Chamberlin, C. to G., Burton-on-Trent. March 23.

Industrial Trainer (Dressmaker) (£26), Portsmouth Guardians. Mr. E. H. Mitchell, C. to G., Portsmouth. March 25.

Infirmary Attendant (£18), Chapel-en-le-Frith Union. Mr. J. B. Boycott, C. to G., Chapel-en-le-Frith. March 23.

Male and Female Attendants on Imbeciles (£26 each), Dudley Union. Mr. E. Allen, C. to G., Dudley. March 26.

Male Lunatic Attendant (£30), Hartlepool Union. Mr. G. Kilvington, C. to G., West Hartlepool. March 26.

Master and Matron (£200 and £90), West Ham Union. Mr. T. Smith, C. to G., Leytonstone, N.E. March 28.

Masters' Clerk for Case-Papers (£25), Southwark Union. Mr. S. Wood, C. to G., Ufford Street, Blackfriars, S.E. March 26.

Master's Clerk for Case-Papers (35s. weekly), Southwark Union. Mr. S. Wood, C. to G., Ufford Street, Blackfriars, S.E. March 27.

Masters' Junior Clerk (9s. weekly), Southwark Union. Mr. S. Wood, C. to G., Ufford Street, Blackfriars, S.E. March 26.

Needlewoman (12s. weekly), Wandsworth Guardians. Mr. F. W. Piper, C. to G., St. John's Hill, Wandsworth, S.W. March 28.

Nurse (£25), Worksop Union. Mr. J. S. Whall, C. to G., Worksop. March 26.

Nurse (£35), Lanchester Union. W. H. Ritson, C. to G., Lanchester, Durham. March 27.

Nurses (3) (£25 each), Richmond Union. Mr. P. Umney, C. to G., Richmond, Surrey. March 28.

Nurses (£26), Doncaster Union. Mr. H. M. Marshall, C. to G., Doncaster.

Nurses (£32), Leigh Union. A. Hayward, C. to G., Union Offices, Leigh, Lancs. April 2.

Porter (£22), Bromyard Union. Mr. F. W. Nicholl, C. to G., Bromyard. March 27.

Porter (£25), Midhurst Union. Mr. J. M. Furneaux, C. to G., Midhurst. March 30.

Portress and Assistant Matron (£25), Newmarket Union. Mr. S. J. Ennion, C. to G., Newmarket. March 27.

Probationer (£15), Braintree Union. A. Hills, C. to G., Union Offices, The Workhouse, Bocking, Essex. March 25.

Probationer Nurse (£10), Hampstead Guardians. H. W. Preston, C. to G., Guardians' Offices, New End, Hampstead. March 25.

Probationer Nurses (£10), Southampton Union. A. J. Walden, C. to G., Workhouse, Southampton.

Probationers (£8), Carlisle Union. Mr. H. B. Lonsdale, C. to G., Carlisle.

Receiving Ward-Mother (£25), Medway Union. Mr. A. R. Norman, C. to G., Chatham. March 23.

Sister (£32), Tynemouth Union. T. Percival, C. to G., Guardians Hall, North Shields. March 30.

